# Long-term management of psoriasis recurrence via modulation of cutaneous microbiome: synergistic topical therapy with blue light and aptamer-functionalized curcumin formulation

**DOI:** 10.1080/10717544.2025.2610532

**Published:** 2026-01-03

**Authors:** Nan Jin, Yaling Chen, Huangyu Luo, Yanhong Su, Yumin Weng, Xin Lin, Tingting Zheng, Bingbing Li, Tianhui Liu, Jianmin Chen

**Affiliations:** a College of Pharmacy and Medical Technology, Putian University, Putian, Fujian, China; b Key Laboratory of Pharmaceutical Analysis and Laboratory Medicine (Putian University), Putian, Fujian, China; c Department of Respiratory and Critical Care Medicine, Affiliated Hospital of Putian University, Putian, Fujian, China; d Department of Respiratory and Critical Care Medicine, Putian Pulmonary Hospital, Putian, Fujian, China

**Keywords:** Psoriasis recurrence, microbiome, blue light, nitric oxide, splenomegaly

## Abstract

The recurrence following the discontinuation of medication is a formidable challenge in managing psoriasis. Changes in the microbiome accompany the onset of psoriasis relapse, highlighting a potential therapeutic modality. To evaluate the superiority of the topical administration of aptamer-functionalized curcumin mesoporous silica (Apt-GA+Cur@μmS) plus blue light (BL) in restoring dysbiosis and intervening in recurrence in a murine model, a psoriasis relapse murine model with double imiquimod induction was established. With a BL-responsive shell, Apt-GA+Cur@μmS released curcumin (Cur) to assist BL to improve the preventative and therapeutic effects in the psoriasis relapse murine model, as evidenced by the psoriasis area and severity index, histology, splenic index, and dorsal IL-17A level. We also observed a negative correlation between splenic nitric oxide (NO) levels and the splenic index, indicating a possible mechanism by which Apt-GA+Cur@μmS&BL may function in the treatment of splenomegaly. Treatment with Apt-GA+Cur@μmS&BL exhibited a higher alpha diversity than the model group, with levels similar to those of healthy mice, indicating that this combination could adjust the composition of the dorsal microbiome to a healthier state. A reduction in the combined relative abundance of *Staphylococcus*, *Streptococcus,* and *Corynebacterium* as well as restoration of dysbiosis was also verified through 16S rDNA gene sequencing *in vivo*. Collectively, BL and Apt-GA+Cur@μmS cotherapy alleviates psoriasiform lesions in a double imiquimod-induced murine model by inhibiting IL-17A and increasing splenic NO. Additionally, this cotherapy restores the eubiosis of the dorsal lesions. Thus, it is a promising and innovative therapeutic modality for psoriasis inflammation alleviation and recurrence intervention.

## 1. Introduction

Psoriasis is a systemic and long-lasting inflammatory disease affecting up to 3% of the global population, with its incidence continuing to increase (Fenix et al. 2020; Lu et al. 2023; Lai et al. 2024). A complex interplay exists between the innate and adaptive immune systems in the pathogenesis of psoriasis (Kamal et al. 2024). Thus, this disease not only results in dermatological disorders characterized by erythematous plaques, scaling, and epidermal hyperproliferation (Lai et al. 2024), but also extends beyond the skin (Kamal et al. 2024). The conventional treatments for psoriasis include phototherapy and immunosuppressive medications, such as methotrexate and cyclosporine (Aziz et al. 2023; Kamal et al. 2024). However, these therapies are limited by their lack of long-term efficacy (Gomes et al. 2023). This is because they focus mainly on alleviating psoriatic dermatitis, reducing inflammatory factors, and prolonging immunosuppression (Aziz et al. 2023; Zhao et al. 2024a), rather than addressing the underlying susceptibility to infection, which is potentially linked to disruptions in the cutaneous microbiome (Lv et al. 2025; Scharschmidt and Segre 2025). Furthermore, upon cessation of treatment following plaque remission (Puig et al. 2022), up to 90% of patients will suffer from recurrence at previously inflamed sites within weeks, months, or even years. This represents one of the most intractable challenges in current psoriasis management (Fenix et al. 2020; Puig et al. 2022; Lu et al. 2023; Deng et al. 2024; Lai et al. 2024). Regrettably, there is currently no definitive cure for psoriasis recurrence (Lai et al. 2024). Therefore, there is an urgent need for long-term therapies.

The precise pathogenesis underlying the susceptibility of psoriasis to relapse following treatment cessation remains elusive. Infection, trauma, and a myriad of other environmental factors have been implicated in this process (Kamal et al. 2024). Recently, one putative mechanism associated with psoriasis recurrence is dysbiosis of the cutaneous microbiome (Chen et al. 2024; Liu et al. 2025a). This is attributed to the crucial role that the microbiota plays in maintaining immune homeostasis, safeguarding against oxidative stress, and defending the body against invading pathogens (Serrage et al. 2024). When dysbiosis occurs, the risk of psoriasis increases, whereas a balanced microbiota may confer protection (Chen et al. 2024). Recent studies (Chang et al. 2018; Lv et al. 2025; Zhao et al. 2024b) have confirmed that the skin microbiota composition of psoriasis patients is heterogeneous compared with that of healthy controls. Thus, the restoration of the cutaneous microbiome represents a promising long-term strategy for managing psoriasis recurrence.

Among the cutaneous microbial inhabitants, *Staphylococcus* (Pan et al. 2025; Scharschmidt and Segre 2025; Liu et al. 2025a), *Streptococcus* (Pan et al. 2025; Liu et al. 2025a), and *Corynebacterium* (Schröder 2024; Liu et al. 2025a) plays important roles in psoriasis progression. Although there are conflicts regarding the influence of single *Streptococcus* (Loesche et al. 2018), single *Staphylococcus* (Chang et al. 2018), or single *Corynebacterium* (Pietrangelo et al. 2023), multiple studies (Alekseyenko et al. 2013; Chang et al. 2018; Loesche et al. 2018; Tao et al. 2022; Lv et al. 2025) have shown that the combined relative abundance of the three bacterial genera in lesional skin is higher than that in healthy people, which seems to be a new paradigm for evaluating the risk of psoriasis recurrence. While some approaches have shown efficacy against individual bacteria *in vitro* (Filippone et al. 2023; Liu et al. 2025b)*,* no therapies have been claimed to inhibit *Staphylococcus*, *Streptococcus,* and *Corynebacterium* simultaneously *in vivo*. Herein, a strategy capable of simultaneously regulating these three bacteria was developed in this study to prevent recurrence.

Targeting IL-17 receptor A (IL-17RA) represents a potential approach to concurrently inhibit *Staphylococcus*, *Streptococcus,* and *Corynebacterium*. First, IL-17A plays a positive role in the host immune response against microbial infections (Lv et al. 2025). Upon the binding of IL-17A (Liu et al. 2024) to IL-17RA or in the absence of IL-17RA (Moos et al. 2023), there is a subsequent reduction in antimicrobial peptides, leading to a weakened defense against the colonization of pathogenic microorganisms, particularly *Staphylococcus*. This, in turn, triggers an increased release of IL-17A. Furthermore, IL-17A synergizes with *Staphylococcus* to promote the expression of other inflammatory cytokines in psoriatic skin lesions (Liu et al. 2025a). Ultimately, this positive feedback loop contributes to a heightened risk of psoriasis relapse. Second, once infected by *Streptococcus*, the number of IL-17A^+^ T cells will be upregulated in psoriatic skin lesions (Zhou et al. 2023), resulting in increased IL-17A production. In addition, *Corynebacterium* stimulates IL-17A production in mouse skin, acting synergistically with IL-23 (Liu et al. 2025a). Consequently, IL-17RA expressed in the skin emerges as a pivotal therapeutic target. Blocking IL-17RA signaling holds promise for restoring antimicrobial defense mechanisms, thereby concurrently suppressing the pathological expansion of *Staphylococcus, Streptococcus,* and *Corynebacterium* and interrupting the vicious cycle driving psoriasis recurrence. However, systemic administration of IL-17A inhibitors could only restore microbial community richness and evenness (Lv et al. 2025), rather than decreasing the sum of the relative abundance of the three taxa, resulting in a tendency to relapse (Lv et al. 2025). Thus, developing an additional strategy to restore the microecological balance is crucial for preventing psoriasis recurrence (Lv et al. 2025).

One promising method to modulate the cutaneous microbiome is by suppressing splenomegaly, which results in abnormal changes in the microbiota composition on the skin of mice (Moos et al. 2023). The enlargement of the spleen size is because of a strikingly imbalance between immune cells (Aziz et al. 2023), and immune imbalance is crucial in exacerbating the relapse of psoriasis (Zhao et al. 2024a). Thus, splenomegaly could act as a primary indicator of immunological responses (Kamal et al. 2024; Serrano et al. 2024) and may be used to identify potential effective candidates to alleviate psoriasis recurrence (Filippone et al. 2023). One possible mechanism of splenomegaly in psoriatic patients is diminished levels of nitric oxide (NO) in the spleen, which results in robust proliferation of splenocytes (Leporati et al. 2024).

NO is a gaseous molecule that can be generated in various types of immune cells (Zaborova et al. 2025) and has been recognized as an important mediator involved in immune systems (Bahadoran et al. 2025; De Abreu Mello et al. 2024; Weitoft et al. 2024). It has demonstrated beneficial effects in vascular-related diseases (Santos-Parker et al. 2017; Abolfazli et al. 2024) and diabetes (Rahiman et al. 2022), both of which are associated with psoriasis recurrence. Even though the function of cutaneous NO has been confirmed to correlate positively with the severity of psoriasis (Man et al. 2022; Alqarni et al. 2025), NO derived from spleen cells is suspected in this study as an agent to suppress the process of psoriasis relapse based on references (De Abreu Mello et al. 2024; Xu et al. 2000; Weitoft et al. 2024), which exhibited that NO could suppress the expansion of primed lymphocytes and inhibit the proliferation of splenocytes, thereby serving as a negative feedback mechanism to limit autoimmune reactions in psoriasis.

Aptamer (Apt) 21-2 (Doble et al. 2014), a single-stranded RNA oligonucleotide, exhibits high specificity and affinity for neutralizing IL-17RA in both humans and mice (Macleod et al. 2019). However, its targeting capability diminishes in the skin environment with abundant antimicrobial peptides, which in turn results in limited efficacy in alleviating dermatitis. Thus, we have previously modified Apt21-2 by conjugating it with glycyrrhizic acid (GA) to develop Apt-GA (Jin et al. 2023). When Apt-GA is loaded with curcumin (Cur) into mesoporous silica in μm-size (μmS), the resulting construct, named Apt-GA+Cur@μmS, is capable of delivering Cur to the dermis of psoriatic lesions as well as inhibiting IL-17A when administered topically after mixing with water (Jin et al. 2023). As a classical antipsoriatic herbal active ingredient, Cur is known for its antioxidant and anti-inflammatory properties (Guarneri et al. 2021), which is attributed to its ability to increase the bioavailability of NO (Abolfazli et al. 2024).

Herein, we propose a novel therapeutic strategy that integrates blue light illumination with the topical administration of Apt-GA+Cur@μmS to modulate aberrant immune responses by balancing cutaneous microbiome colonization, thereby preventing psoriasis recurrence. Blue light is selected because of its anti-microbial and anti-inflammatory properties (Serrage et al. 2024) through self-produced NO without causing significant tissue damage (Sadowska et al. 2021). We hypothesize that after Apt-GA+Cur@μmS reaches the skin target expressing IL-17RA, upon irradiation with blue light, the synergistic effect of blue light therapy and topical administration of Apt-GA+Cur@μmS would alleviate splenic NO-mediated splenomegaly as well as reduce IL-17A expression in the skin. These effects would diminish the abundance of cutaneous *Staphylococcus*, *Streptococcus,* and *Corynebacterium* within a murine model of psoriasis relapse provoked by imiquimod (IMQ), compared to either strategy employed alone.

## 2. Materials and methods

### 2.1 Materials

Curcumin (M = 368.38, AR, purit98%, D9053003) was purchased from Shanghai SunnyBiotech Ltd. (Shanghai, China). Glycyrrhizic acid (M = 822.93, purity >  95%, P1850507) were purchased from Shanghai Titan Technology Co., Ltd. (Shanghai, China). The sequence of this Apt is 5'-NH_2_-G-G-fU-fC-fU-A-G-fC-fC-G-G-A-G-G-A-G-fU-fC-A-G-fU-A-A-fU-fC-G-G-fU-A-G-A-fC-fC-3'-inverted dT synthesized by Sangon Biotech Ltd. (Shanghai, China). The microsized mesoporous silica (μmS) Aeroperl^®^ 300Pharm (Lot: 156062219) was a gift from Evonik Industries AG (Peking, China). Griess reagent (Lot: PLM053) was purchased from Fuzhou Phygene Life Sciences Co., Ltd. (Fuzhou, China). Dimethyl sulfoxide (DMSO, AR grade, F2217341) was purchased from Shanghai Aladdin Bio-Chem Technology Ltd. (Shanghai, China). A Dura 12 water purification system (The Lab Instruments and Technology Ltd., Shanghai, China) was used to obtain freshly produced ultra-purified water. Stroke-physiological saline solution (M = 58.44, 0.9% NaCl, pH 7.0, JR25548A) was purchased from Yuanye Bio-Technology Co., Ltd. (Shanghai, China). Luria-Bertani Broth (Lot: GPG2407004) was purchased from Wuhan Servicebio Technology Co., Ltd. (Wuhan, China). A mouse IL-17A ELISA kit (Lot: 230919 A) was obtained from Hunan Youbaihui Biotechnology Co., Ltd. (Changsha, China).

### 2.2 Animals

Five-week-old female BALB/c mice were purchased from Hangzhou Ziyuan Laboratory Animal Technology Co., Ltd. (Hangzhou, China) with the license No. 20240719Abbz0105000654. Each mouse was housed in a single cage furnished with Corn Cob bedding. After acclimatizing for 1 week, six mice were randomly selected from each group. For each group, six different investigators were involved as follows: investigators A and B administered the treatment based on the design, investigator C, D, and E were responsible for the detection, and investigator F analyzed the data. The mice were subjected to the designed protocols, which were conducted in strict compliance with the laboratory animal care guidelines and received approval from the Ethics Committee of the Affiliated Hospital of Putian University (Putian, China, 2025DW069).

### 2.3 Physical characterization

The formulations entitled Apt-GA + Cur@μmS, GA + Cur@μmS and Cur@μmS were fabricated through a four-step process as detailed in a previous publication (Jin et al. 2023), which is outlined below briefly. First, the RNA aptamer labeled as Apt (MW 11196 Da) was conjugated to GA via an EDC/NHS reaction to obtain Apt-GA. Second, Apt-GA or GA was mixed with Cur in a 8:1 (w/w) ratio. The mixture or Cur alone was then dissolved in DMSO to achieve a Cur/DMSO solution (1.32.5 mg/mL). Third, the Cur-containing DMSO solution was sprayed onto μmS at a weight ratio of 1:50. Finally, the removal of DMSO was conducted at 65 °C in a vacuum drying oven.

Cur RDP, Cur@μmS, GA + Cur@μmS, and Apt-GA + Cur@μmS with 0.42  mg Cur were added to 350  μL MilliQ-water in a 6-cell plate (120  rpm, 37 °C) to mix thoroughly. In order to achieve sink condition, 350  μL of DMSO was also added into the cell. Eight  hours later, the mixture was exposed to 450 nm blue light (130 mw/cm^2^) for 1  min in a 6-cell plate. A total of 50  μL of the supernatant was diluted with 950  μL MilliQ-water for subsequent observation. The morphology of the supernatant was performed by the fluorescence microscope (OLYMPUS, BX53) equipped with a U-FBW laser module and a 515 nm longpass filter (excitation of 450~480 nm). The particle size distribution of the supernatant was measured via the dynamic light scattering method using photon correlation spectroscopy (PCS, Zeta Size Nano ZS90, Malvern Panalytical, UK). The supernatant was subsequently transferred to a U-tube cuvette (DTS1070, Malvern) to measure the zeta potential.

### 2.4 Establishment of IMQ double-induced psoriasis relapse mouse model and treatment

**Figure 1.**
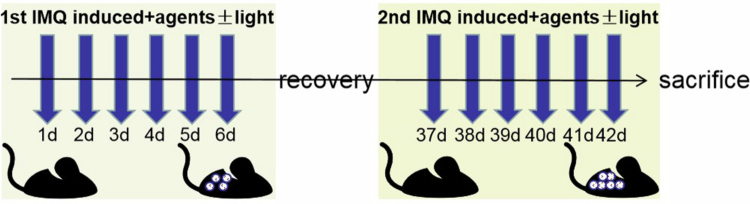
Treatment schedules for the double IMQ-induced psoriasis recurrence investigation. Four hours after the topical administration of IMQ, Apt-GA+Cur@μmS was administered topically with ultra-purified water daily from day 1 to day 6 and from day 37 to day 42, 0.5 h prior to blue light irradiation or without blue light irradiation.

The dorsal skin (2  cm × 3  cm) of mice was depilated after using a depilatory cream and a shaver, which was recorded as day 0. Psoriasis relapse models were established according to the methodology outlined in the previous study (Liu et al. 2025b) with minor modifications as follows. All the mice, with the exception of those in the blank group, were administered 62.5  mg of 5% IMQ cream per mouse topically on the depilated area once daily during the initial 6 days and the last 6 days, as displayed in Figure 1. This prediction of psoriasis relapse was first validated through optical observation of signs of psoriatic skin inflammation, including erythema, scales, and thickness on the dorsal side of the skin. These signs were scored separately according to the Psoriasis Area and Severity Index (PASI). The scores were separately as 0, 1, 2, 3, and 4. The higher the score is, the greater the severity. The cumulative score, which is calculated by summing the three indices and ranges from 0 to 12, is employed to measure the level of inflammation associated with the skin condition resembling psoriasis. Additionally, the prediction was also validated through histopathological analysis using hematoxylin and eosin (H&E) staining.

The various treatment regimens were applied 4 h after the application of IMQ cream. The model group received no additional treatment, whereas the model&BL group was exposed to blue light (450 nm) from above the dorsal skin 20  cm for 3 min (511 mw/cm^2^). The Apt-GA+Cur@μmS group was treated with 0.42  mg Cur/day/mice with Apt-GA+Cur@μmS, followed by illumination (light+) or not (light−). Twenty-four  hours after the final day of treatment, the mice underwent scarification through cervical dislocation, and skin tissue samples were collected for further analysis.

### 2.5 Immune imbalance

Spleen tissues were excised from six mice in each group at day 43. After the removal of the fascia and surrounding adipose tissue, the weight and the length of each spleen were measured. The weight of the spleen to the body weight ratio was calculated to assess the splenomegaly as previously reported (Aziz et al. 2023).

### 2.6 NO colorimetric assay

Secreted NO quickly reacts with oxygen to yield nitrite, herein levels of nitrite were measured as an indicator of NO production by a colorimeter assay based on the Griess reaction (He et al. 2024; Alqarni et al. 2025). The secreted NO was detected *in vitro* and *in vivo*, respectively. For *in vitro* detection, Apt-GA+Cur@μmS with 0.42  mg Cur inside was added to 350  μL MilliQ-water in a 6-cell plate (120  rpm, 37 °C) for thorough mixing. To achieve sink conditions, 350  μL of DMSO was also added into the cell. Eight hours later, those mixture was exposed to 450 nm blue light (511 mw/cm^2^) for 3  min in a 6-cell plate. The mixture was then heated at 100 °C for 5  min to eliminate the color influence of Cur. A total of 100  μL of the supernatant was diluted by 900  μL MilliQ-water for subsequent mixing with Griess reagent. After another incubation for 30  min, the absorbance was measured at 450 nm in an automated plate reader.

For *in vivo* detection, the spleen was isolated and homogenized according to our published method (Jin et al. 2025). The collected spleen was mixed with 9 times the amount of PBS (pH 7.2, 0.01 M) and homogenized via a tissue homogenizer (KZ-III-F, Servicebio, Wuhan) at 4 °C for 4 min. The supernatant was obtained via centrifugation (21100 × g, 4 °C, 20 min). A total of 10  μL of spleen supernatant was mixed with 50  μL of saline and 40  μL of Griess reagent. After incubating for 30  min at room temperature, the absorbance was measured at 450 nm in an automated plate reader.

### 2.7 Enzyme-linked immunosorbent assay (ELISA)

The plaque-affected dorsal skin of 6  cm^2^ size from IMQ-induced mice was isolated and cut into pieces, followed by homogenized as previously described (Jin et al. 2025). In brief, the skin sample was combined with 9 times the volume of PBS (pH 7.2, 0.01 M) and homogenized using a tissue homogenizer (KZ-III-F, Servicebio, Wuhan) at 4 °C for 4 min. The sample was subsequently centrifuged at 21,100 × g at 4 °C for 20 min to obtain the supernatant. IL-17A in the supernatant were quantitated through ELISA following the manufacturers' instructions. The OD450 was measured with the plate photometer reader (F50, Tecan).

### 2.8 Microbiome analysis

We further investigated the influence of Apt-GA+Cur@μmS on the dorsal microbiota of the mice. Skin samples were collected as published method (Liu et al. 2024a, 2024b) for microbiome analysis as follows. The dorsal skin of each mouse was rinsed with sterile saline to obtain the bacterial rinse fluid after agent application for at least 24 h. The bacterial rinse fluid collected from each group was spread onto agar plates with the streak plate method and cultured in a constant-temperature incubator at 37 °C. After 24 h, bacterial colonies were counted. Statistical differences in colony counts were analyzed by the Mann–Whitney U.

Skin swabs were taken from the center area of the dorsal lesion, whose diameter range is 1  cm. Each swab was spread in a sterile cryopreservation tube with 2 mL of sterile saline, and was frozen into liquid nitrogen immediately and stored at -80 °C for further DNA extraction. The 16S rDNA high-throughput sequencing technology was then performed following a previously reported protocol (Alekseyenko et al. 2013; Liu et al. 2024a) to analyze the composition and abundance of cutaneous bacterial communities in mice. In brief, PCR was conducted using universal primers 341F (5'-CCTAYGGGRBGCASCAG-3') and 806 R (5'-GGACTACNNGGGTATCTAAT-3') to amplify the V3-V4 variable regions of the bacterial 16S rRNA gene. The final amplicon was quantified using the NEBNext Ultra II DNA Library Prep Kit (Cat. No. E7645B) from New England Biolabs. Quality filtering on the raw tags was performed using the fastp (Version 0.23.1) software to obtain high-quality clean tags. The tags were compared with the reference database (Silva138.1 database (16S/18S), https://www.arb-silva.de/; Unite Database (ITS), https://unite.ut.ee/) to detect chimera sequences. The effective tags were obtained by removing the chimera sequences with the vsearch package (V2.16.0, https://github.com/torognes/vsearch).

The data was analyzed using the published method (Moos et al. 2023), which is briefly described below. Alpha diversity, which encompasses richness and evenness, was employed to analyze the species diversity within a group. This was achieved through four indices, including the observed operational taxonomical units (OTU), Shannon index, Simpson index, Chao 1 index, and Pielou_e index. To evaluate the complexity of the community composition and compare differences between groups, beta diversity was calculated using weighted and unweighted UniFrac distances in QIIME2, where principal coordinate analysis (PCoA) was used to analyze the similarities and differences in the composition of the microbiota. The samples with significant community differences were positioned further apart. Relative abundance comparisons of the top 10 taxa were made at both the phylum and genus levels for each sample, and the distribution histogram of relative abundance was plotted in Perl through the SVG function.

### 2.9 Statistical analysis

All studies were repeated at least three times. Student's *t*-test was used to evaluate data with a normal distribution, while Mann-Whitney *U* test was used to evaluate data with an abnormal distribution. The disparity in alpha diversity across groups was scrutinized employing the Kruskal‒Wallis test. All the statistical analyzes were performed with a confidence interval of 95%. *P*-values < 0.05 were considered statistically significant.

## 3 Results and discussion

### 3.1 Physical characterization

**Figure 2.**
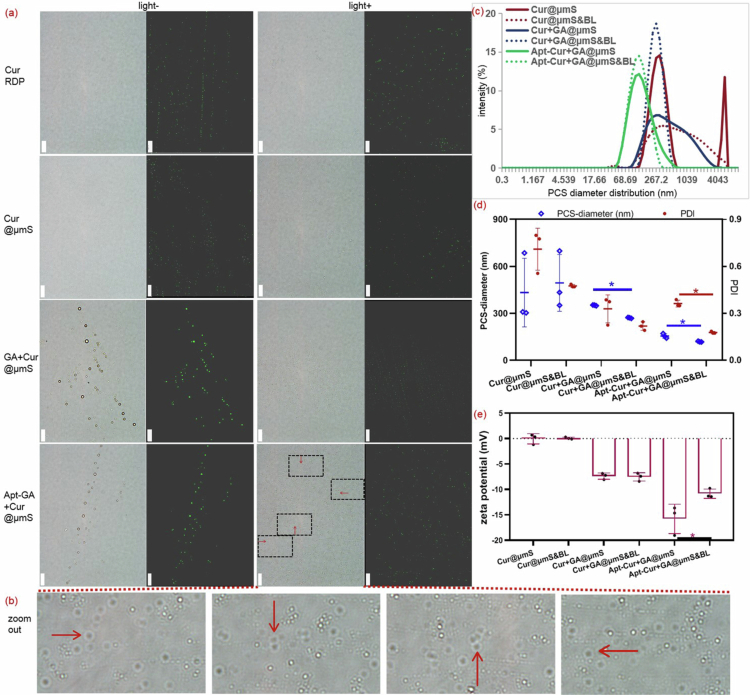
The influence of Apt-GA on the physical characteristics of Apt-GA+Cur@μmS. (a) Optical microscopical photographs and fluorescent photographs of Apt-GA+Cur@μmS and its controls before (light−) or after (light+) blue light irradiation (1000×, scale bar = 10 μm). (b) The enlarged version of the microscopical photograph of Apt-GA+Cur@μmS after blue light irradiation. (c) Representative hydrodynamic diameter distribution of Apt-GA+Cur@μmS and its controls before or after blue light (BL) irradiation. (d) Average photon correlation spectroscopy (PCS)-diameter and PDI of Apt-GA+Cur@μmS and its controls before or after blue light irradiation (*n* = 3, mean ± SD, Student's *t*-test, ^*^P < 0.05). (e) Average zeta potential of Apt-GA+Cur@μmS and its controls before or after blue light irradiation (*n* = 3, mean ± SD, Student's *t*-test, ^*^P < 0.05).

As depicted in Figure 2a, yellow circles are visible in the supernatants of both GA+Cur@μmS and Apt-GA+Cur@μmS groups under an optical microscope before exposure to blue light. Correspondingly, green fluorescent circles are superimposed with those yellow circles when measured under a fluorescence microscope. In contrast, only yellow dots and green fluorescent dots were observed in the supernatants of Cur RDP and Cur@μmS. These observations suggest that Apt-GA and GA probably function as amphiphilic materials to form micelles that encapsulate Cur. After exposure to blue light irradiation, the circles of both GA+Cur@μmS and Apt-GA+Cur@μmS transformed into dots similar to those of Cur RDP and Cur@μmS. Furthermore, an unexpected phenomenon of shell shedding was discovered in the Apt-GA+Cur@μmS group, as shown by the red arrow (zoomed out in Figure 2b). This finding indicates that upon mixing Apt-GA+Cur@μmS with water, Apt-GA may self-assemble into micelles containing Cur, where the release of Cur is controlled by the illumination of blue light.

In addition to the results of morphological observation, the particle size distribution (Figure 2c,d) measured using dynamic light scattering method further confirmed the differences among Cur@μmS, GA+Cur@μmS, and Apt-GA+Cur@μmS. Large particles close to 4000 nm in size were detected in Cur@μmS, resulting in an average size of 432.9 ± 219.3 nm and a particle size distribution (PDI) > 0.7. While an average size of 155.6 ± 14.8 nm and a PDI of 0.363 ± 0.022 were detected in Apt-GA+Cur@μmS. Moreover, following blue light illumination, the intensity of both Apt-GA+Cur@μmS and GA+Cur@μmS increased (Figure 2c). In contrast, the intensity of Cur@μmS decreased after exposure to blue light. These phenomena suggest that Cur nano-micelles could be generated by adding Apt-GA or GA during the preparation of Cur@μmS, whose release may be controlled through blue light irradiation. Although the average PCS-diameter (Figure 2d) of the supernatant of both GA+Cur@μmS and Apt-GA+Cur@μmS was between 50 and 500 nm, which facilitates penetration into the skin (Niu et al. 2022), Apt-GA+Cur@μmS is more prone to delivering Cur to the deep layers of the skin since its particle size is less than 300 nm (Niu et al. 2022).

The differences in zeta potential data (Figure 2e) are observed only between the formulations with and without blue light irradiation for Apt-GA+Cur@μmS (*P* < 0.05), not for GA+Cur@μmS (*P* > 0.05) and Cur@μmS (*P* > 0.05). This finding is analogous to the morphological observation (Figure 2a), indicating that irradiation with blue light aids in the decortication of Apt-GA from Cur. Besides, Apt-GA is imperative for proper colloidal particles, as its absolute surface charge value is greater than that of GA+Cur@μmS (*P* < 0.05) and Cur@μmS (*P* < 0.05), indicating superior stability (Alqarni et al. 2025).

### 3.2 Establishment of IMQ double-induced psoriasis relapse mouse model and treatment

**Figure 3.**
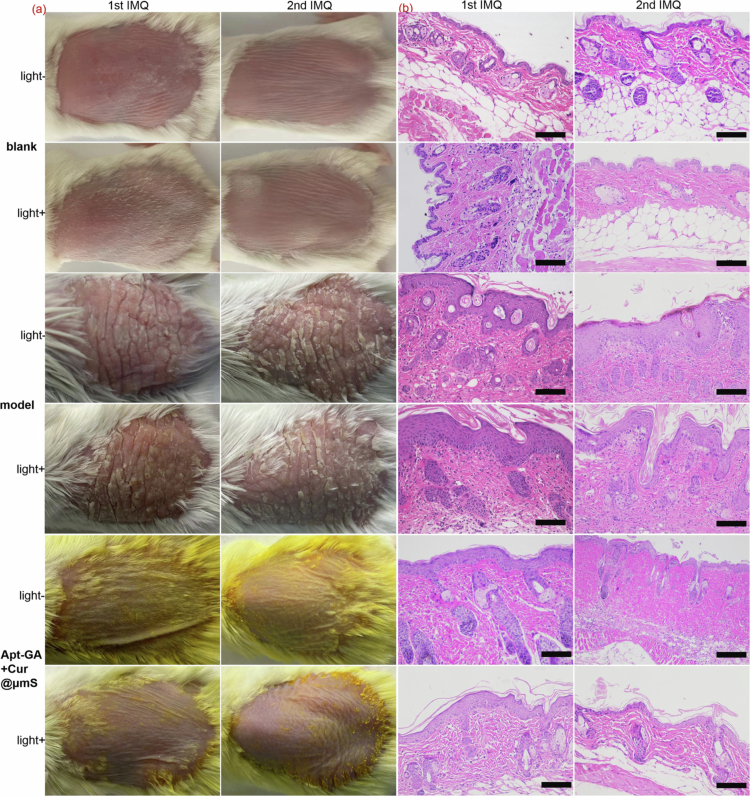
Evaluation of effectiveness of combined Apt-GA+Cur@μmS and blue light irradiation therapy on dermal symptoms within a murine model induced by the first round of IMQ and the second round of IMQ. (a) Representative appearance of lesions. (b) Representative H&E staining images of dorsal skin lesions (200×, scale bar = 100 μm).

**Figure 4.**
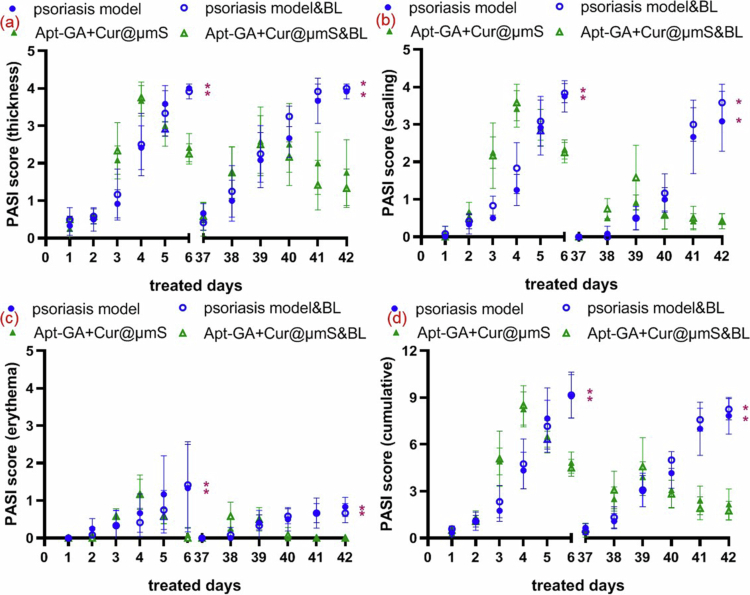
PASI scores of the murine model induced by the first round of IMQ and the second round of IMQ after different treatments (*n* = 6, mean ± SD, Student's *t*-test, ^*^P < 0.05 compared to Apt-GA+Cur@μmS&BL group). (a) PASI scores on the thickness. (b) PASI scores on the scaling. (c) PASI scores on the erythema. (d) Cumulative PASI scores.

The IMQ-induced psoriasis model is a common tool for evaluating therapeutic effects because it not only phenocopies skin features of psoriasis but also has similar gene expression profiles as those of psoriatic patients (Moos et al. 2019). As depicted in Figures 3a and 4, topical application of IMQ cream provoked extensive erythema, characterized by redness of the inflamed back (Filippone et al. 2023), flaky scaled skin and enhanced skin thickness compared to the blank group. Notably, the average score for erythema induced by IMQ is less than 2 (Figure 4c), while that for either thickness (Figure 4a) or scaling (Figure 4b) is greater than 3. A similar phenomenon has also been reported in other psoriasis relapse models (Liu et al. 2025b, 2025c), which are both induced by IMQ. Thus, the knowledge of the weak induction of erythema, in our opinion, is imperative for the proper improvement of the psoriasis relapse model protocol. Since *Corynebacterium* genus is the microbial determinant of erythrasma (Pietrangelo et al. 2023), concern of *Corynebacterium* was conducted in this study.

The topical treatments of Apt-GA+Cur@μmS, with or without blue light irradiation, both relieve the psoriasis-like phenotypes within the psoriasis relapse murine model macroscopically (Figure 3a) and microcosmically (Figure 3b). After treatment, histological features of the skin layers (Figure 3b), including a thinner epidermal layer and less infiltration of inflammatory cells, were observed. These findings suggest that Apt-GA+Cur@μmS can remarkably reduce excessive inflammation in psoriasis patients and aid in relapse prevention. The addition of blue light did not show superiority on the alleviation of psoriasis-like dermatitis.

### 3.3 Immune imbalance

**Figure 5.**
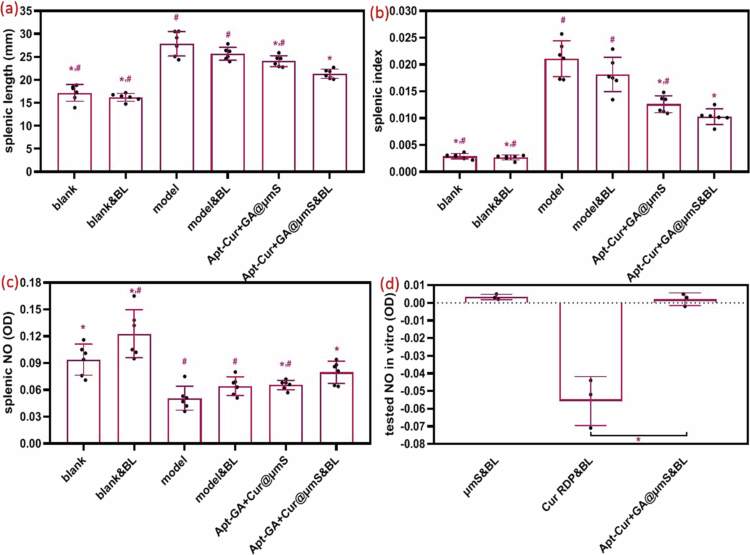
Splenic characteristics of the excised spleen of the murine model after different treatments on day 43 (*n* = 6, mean ± SD, Student's *t*-test, ^*^P < 0.05 compared to model group, ^#^*P* < 0.05 compared to Apt-GA+Cur@μmS&BL group) and detected NO (*n* = 3, mean ± SD, Student's *t*-test, ^*^P < 0.05). (a) The length of the spleen in the different groups. (b) The splenic index of different groups. (c) The detected NO level in the spleen. (d) The detected NO level in the 96-cell plate.

The results demonstrated a pronounced increase in both the splenic length (Figure 5a) and the spleen index (Figure 5b) after the topical application of IMQ cream (Kamal et al. 2024). The attenuation of the enlarged splenic length and spleen index (*P* > 0.05) could not be achieved by single blue light but rather by the combination with Apt-GA+Cur@μmS (*P* < 0.05). A significant disparity was even evident in the splenic length and spleen index between the Apt-GA+Cur@μmS&BL and Apt-GA+Cur@μmS group (*P* < 0.05). This finding indicates that the addition of blue light may modulate these heightened immune reactions.

### 3.4 NO colorimetric assay

NO has been considered a double-edged sword, mediating tissue damage on the one hand while modulating complex immunoregulatory functions on the other hand (Xu et al. 2000; Meki and Al‐Shobaili 2014). Most of the studies aimed to determine the function of NO in psoriatic plaques (Meki and Al‐Shobaili 2014) or serum (Meki and Al‐Shobaili 2014) or macrophages (He et al. 2024) or endothelium (Alba et al. 2018) or spleen (Xu et al. 2000). However, seldom strategies have been reported to alleviate psoriasis by increasing splenic NO. The effect of treatment on NO levels *in vitro* or in the spleen was detected by the Griess reaction in this study. As shown in Figure 5, the splenic NO level (Figure 5c) is increased significantly (*P* < 0.05) after the treatment of Apt-GA+Cur@μmS with or without blue light irradiation. The combination therapy is even superior to the one without the assistance of blue light (*P* < 0.05). Surprisingly, the splenic NO level (Figure 5c) was negatively correlated with the splenic index (Figure 5b) and splenic length (Figure 5a). On the contrary, seldom NO was detected *in vitro* (Figure 5d). Based on the result expressed in Figure 2, one suspicion is that the micelle with Cur inside would take off the shell, which is composed of Apt-GA under the stimulation of blue light, following the penetration into the dermis with the targeting capability of Apt-GA (Jin et al. 2023). The ROS are then be produced (Jin et al. 2025), consuming the accumulated L-arginine in skin lesions to NO (Li et al. 2024; Miao et al. 2024), which is transferred to the spleen through blood circulation. Secreted NO suppresses the expansion of primed lymphocytes (Xu et al. 2000), thereby restricting autoimmune responses in psoriasis patients (Alba et al. 2018) and relieving the splenomegaly. Furthermore, the OD value of the tested NO in the Cur RDP&BL group (Figure 5d) was lower than 0, indicating that the structure of Cur was broken when exposed to blue light without any formulation protection. Based on the results shown in Figures 2 and 5, the formulation Apt-GA+Cur@μmS was demonstrated to be capable of being stimulated by blue light, producing splenic NO to initiate psoriasis relapse.

### 3.5 ELISA

**Figure 6.**
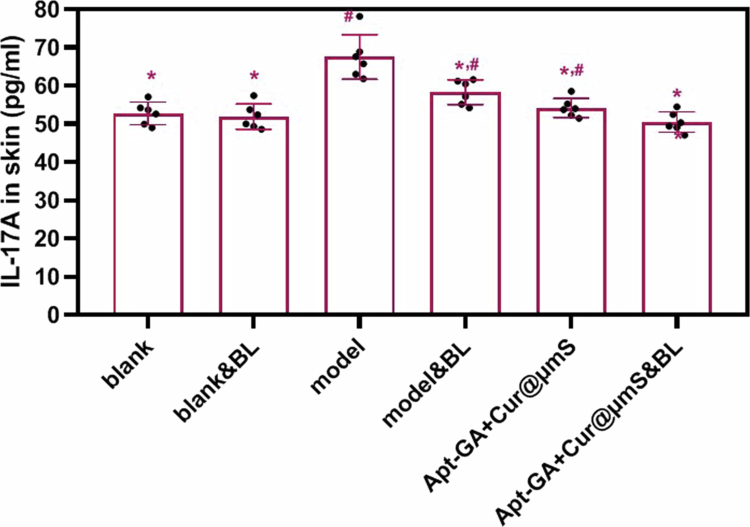
Effect of different treatments on dorsal IL-17A level (*n* = 6, mean ± SD, ^*^P < 0.05 compared to model group, ^#^*P* < 0.05 compared to Apt-GA+Cur@μmS&BL group).

The ELISA data (Figure 6) indicated that both blue light irradiation and topical administration of Apt-GA+Cur@μmS significantly reduced the expression of IL-17A in the dorsal skin lesions of psoriasis relapse model mice (*P* < 0.05). However, the IL-17A level in the combined therapy group was lower (*P* < 0.05). Thus, combination treatment can repress IL-17A related to psoriasis onset and development, which is superior to either single strategy, as expected.

### 3.6 Microbiome analysis

**Figure 7.**
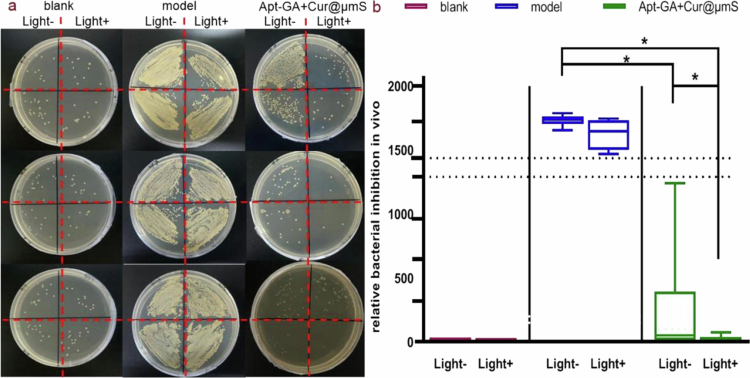
*In vitro* antibacterial effects on dorsal skin of the murine model after different treatments on day 43 (*n* = 6, median with range, Mann–Whitney *U* test, ^*^P < 0.05). (a) All images of plate colonies. (b) Corresponding colony count statistics.

The plate colony counting method was employed to assess the antibacterial efficacy of the combined therapy. The colony counts (Figure 7) clearly indicated that Apt-GA+Cur@μmS&BL exhibits inhibitory effects on dorsal bacteria, which are stronger than those of Apt-GA+Cur@μmS and blue light (*P* < 0.05). Blue light alone did not demonstrate any significant antibacterial effect (*P* > 0.05). The number of colonies in the Apt-GA+Cur@μmS&BL group (21 ± 29 CFU) was even similar to that of the blank group (17 ± 5 CFU), suggesting that the combination strategy is an effective therapeutic modality for restoring the microbiome of the dorsal skin, much better than single Apt-GA+Cur@μmS treatment or blue light irradiation.

**Figure 8.**
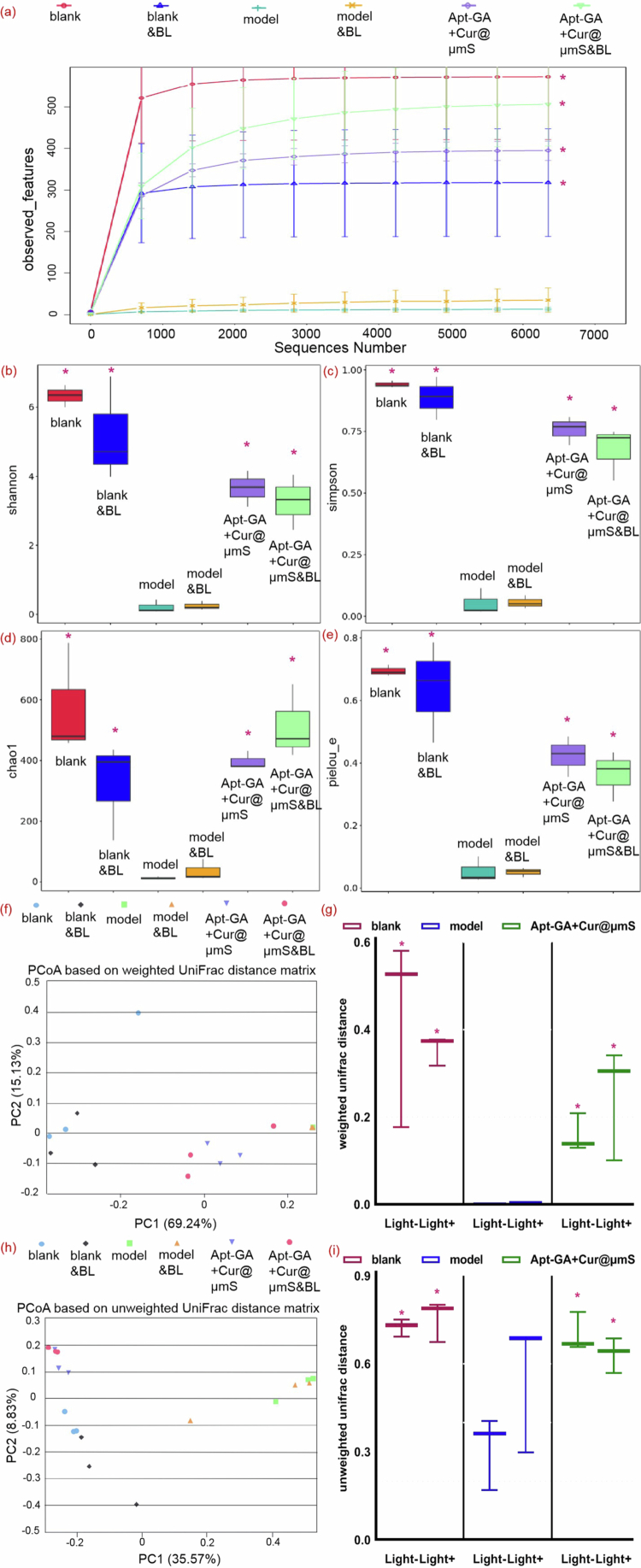
Effects of each group on the composition of mouse dorsal skin microbiota in alpha diversity (Kruskal–Wallis test) and beta diversity (*n* = 3, ^*^P < 0.05 compared to model group). (a) Species diversity curves for each group (median with the upper limit of the species richness estimate, and with the lower limit). (b) Alpha-diversity index of Shannon (median with range). (c) Alpha-diversity index of Simpson (median with range). (d) Alpha-diversity index of Chao 1 (median with range). (e) Alpha-diversity index of Pielou_e (median with range). (f) PCoA of beta diversity of weighted UniFrac distances. Each point represents a single sample that is color-coded according to group, and the two principal components (PC1 and PC2) explained 69.24% and 15.13%, respectively. (g) The average weighted UniFrac distances among samples within each group are displayed in the box plot (median with range, Student's *t* test). (h) PCoA of beta diversity of unweighted UniFrac distances. Each point represents a single sample that is color-coded according to group, and the two principal components (PC1 and PC2) explained 35.57% and 8.83%, respectively. (i) The average unweighted UniFrac distances among samples within each group are displayed in a box plot (median with range, Student's *t*-test).

The microbiota of the untreated psoriasis relapse group (model) was compared with that of the Apt-GA+Cur@μmS&BL group to analyze microbiota components influenced by the combined therapy. Double induction of IMQ (model) caused remarkably alterations in the alpha- and beta-diversity of the microbiota on the skin (Figure 8 a-i), suggesting greater heterogeneity, as reported in psoriatic patients (Lv et al. 2025; Zhao et al. 2024a). After the administration of Apt-GA+Cur@μmS&BL, the Kruskal‒Wallis test expressed notable differences in the observed OTUs (Figure 8a), Shannon index (Figure 8b), Simpson index (Figure 8c), Chao 1 index (Figure 8d), and Pielou_e index (Figure 8e). These alpha diversity analysis data demonstrated that the bacterial diversity of the Apt-GA+Cur@μmS&BL group was significantly higher than that of the model group, which indicates more stable ecosystems, followed by healthier skin conditions (Lv et al. 2025).

Consistent results were also observed in the PCoA results that utilized both weighted (Figure 8f) and unweighted (Figure 8h) UniFrac distances. These findings indicate that the Apt-GA+Cur@μmS&BL group exhibited a lower mean distance in the composition of their dorsal microbiota compared to the model group, suggesting decreased heterogeneity and closer proximity to that of the blank group. All the results indicate a restoration of skin microbial community richness and evenness post-treatment with Apt-GA+Cur@μmS&BL, which parallels the findings of research (Lv et al. 2025) that compared the skin microbiota of psoriasis patients before and after the intravenous administration of an IL-17A inhibitor.

**Figure 9.**
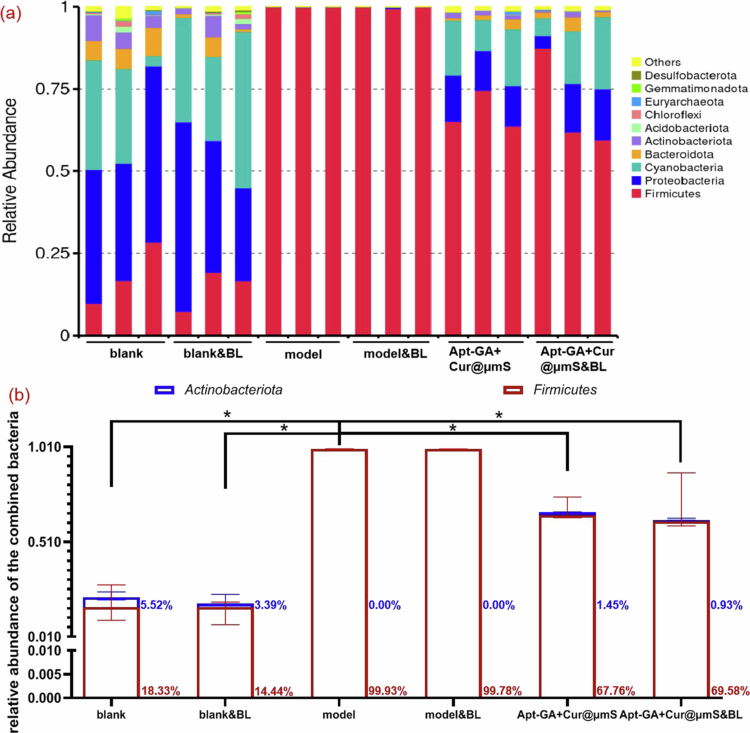
Relative abundance of bacteria at the phylum level in the mouse dorsal microbiota (*n* = 3, median with range, Mann‒Whitney *U* test, ^*^P < 0.05). (a) Composition of the microbiota in each sample. (b) Combined relative abundance of *Firmicutes* and *Actinobacteriota*.

Moreover, it has been reported (Lv et al. 2025) that *Proteobacteria* is the dominant phylum in the groups of healthy people. The same phenomenon also occurs in the blank mice in this study (Figure 9a). In contrast, *Actinobacteriota* constituted the highest proportion of the microecological level in psoriasis patients, both before and after treatment with IL-17A inhibitors (Lv et al. 2025), while *Firmicutes* was the most abundant phylum in the IMQ double-induced psoriasis relapse mouse model with or without treatment in this study (Figure 9a). A similar finding has also been reported in other research (Zhao et al. 2024a), which detected the major phylum *Firmicutes* on the mouse model through 7 days of IMQ stimulation. One possible explanation for the varying abundance of phyla is that *Actinobacteriota* was identified in patients diagnosed with psoriasis vulgaris (Lv et al. 2025), whereas the administration of IMQ in this study induced plaque-type psoriasis (Zeini et al. 2021), in which the dominant microbiome in patients belongs to the phylum *Firmicutes* (Assarsson et al. 2018).

**Figure 10.**
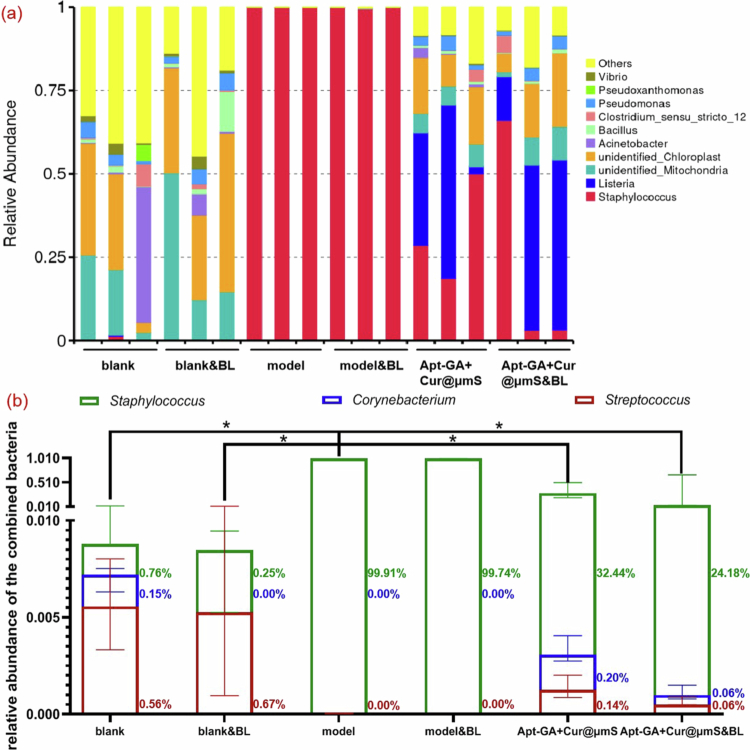
Relative abundance of bacteria at the genus level in the mouse dorsal microbiota (*n* = 3, median with range, Mann‒Whitney *U* test, ^*^P < 0.05). (a) Composition of the microbiota in each sample. (b) Combined relative abundance of *Staphylococcus*, *Corynebacterium*, and *Streptococcus*.

As shown in Figure 10a, *Staphylococcus* was the most abundant cutaneous genus in the model group, similar to other reports (Shinno-Hashimoto et al. 2021; Zhao et al. 2024a). Compared with blank lesions, significantly higher combined relative abundances of *Staphylococcus*, *Streptococcus,* and *Corynebacterium* at the genus level were observed in psoriatic lesions (Figure 10), suggesting that the enhanced combined relative abundances of the three genera contribute to psoriasis recurrence (Alekseyenko et al. 2013). The results also showed a lower combined relative abundance of the three genera in the lesions of the Apt-GA+Cur@μmS&BL group compared to that of the model group. The findings indicated a negative correlation between the combined relative abundance and symptoms resembling psoriasis. In addition, *Staphylococcus* was the dominant microbiota at the genus level (Figure 10), which is consistent with observations on the dorsal skin of an acute psoriasis murine model induced by IMQ (Shinno-Hashimoto et al. 2021) and psoriasis patients (Filippone et al. 2023). As expected, *Corynebacterium* was seldom detected in the model group, contributing to the slight erythrasma (Pietrangelo et al. 2023) of psoriasis relapse model shown in Figure 4c. Extra *Corynebacterium* will be added to the dorsal region of the murine model to exacerbate psoriasis-like symptoms in the future.

## 4. Conclusions

This study revealed that the combination therapy of blue light and Apt-GA+Cur@μmS is an alternative and valid treatment for delaying psoriasis recurrence. It alleviated splenomegaly, decreased IL-17A levels, and improved the stability of the microbial community, which are all much superior to single blue light therapy. Furthermore, it diminished the combined relative abundances of three genera, *Staphylococcus*, *Streptococcus,* and *Corynebacterium*, on the dorsal skin. Finally, we also elucidated that the mechanism of the combined therapy on splenomegaly is very likely through NO. Despite these promising results, the low number of mice included in the 16S rDNA high-throughput sequencing investigation necessitates scaling up the study to include more samples to confirm our findings.

## Supplementary Material

Supplementary Material 2_ARRIVE_guidelines_Author_Checklist_E10_only_20251121.pdf

## Data Availability

The data will be provided by the authors on request.
